# Neighborhood deprivation and midlife cognition: Evidence of a modifiable vascular pathway involving health behaviors and cerebral small vessel disease

**DOI:** 10.1002/alz.70756

**Published:** 2025-11-05

**Authors:** Audrey Low, Kamen A. Tsvetanov, Georgios Ntailianis, Maria A. Prats‐Sedano, Elizabeth McKiernan, Stephen F. Carter, James D. Stefaniak, Stefania Nannoni, Li Su, Anna McKeever, Maria‐Eleni Dounavi, Graciela Muniz‐Terrera, Katie Bridgeman, Sarah Gregory, Karen Ritchie, Brian Lawlor, Lorina Naci, Charlotte Connolly, Paresh Malhotra, Ivan Koychev, Craig W. Ritchie, John T. O'Brien, the PREVENT Dementia Investigators

**Affiliations:** ^1^ Department of Psychiatry School of Clinical Medicine Cambridge Biomedical Campus University of Cambridge Cambridge UK; ^2^ Department of Radiology Mayo Clinic Rochester Minnesota USA; ^3^ Department of Psychology University of Cambridge Cambridge UK; ^4^ Department of Clinical Neurosciences Cambridge Biomedical Campus University of Cambridge Addenbrooke's Hospital Cambridge UK; ^5^ Edinburgh Dementia Prevention University of Edinburgh Edinburgh UK; ^6^ Department of Neuroscience University of Sheffield, Western Bank Sheffield UK; ^7^ Heritage College of Osteopathic Medicine Ohio University Athens USA; ^8^ Scottish Brain Sciences Edinburgh UK; ^9^ Research Unit 1061 (Neuropsychiatry) INSERM Montpellier France; ^10^ Institute of Neuroscience Trinity College Dublin College Green Lloyd Building Trinity College Dublin Ireland; ^11^ Division of Brain Sciences Imperial College Healthcare NHS Trust Hammersmith Hospital London UK; ^12^ Department of Psychiatry University of Oxford Warneford Hospital Oxford UK; ^13^ Brain Health and Neurodegenerative Medicine Mackenzie Institute University of St Andrews St Andrews UK; ^14^ Cambridgeshire and Peterborough NHS Foundation Trust Elizabeth House Fulbourn Hospital Cambridge UK

**Keywords:** brain health disparities, cerebral small vessel disease, dementia prevention, midlife cognition, modifiable risk factors, neighborhood deprivation, structural determinants of health, vascular cognitive impairment

## Abstract

**INTRODUCTION:**

Neighborhood deprivation increases dementia risk, although mechanisms remain unclear. We tested a framework in which modifiable risk factors and cerebral small vessel disease (SVD) mediate the link between neighborhood deprivation and cognition.

**METHODS:**

In 585 cognitively healthy midlife adults (ages 40–59), neighborhood deprivation was derived from postcodes, cognition was assessed using the COGNITO, lifestyle risk factors were measured using clinical assessments, and SVD (white matter hyperintensities, lacunes, microbleeds, perivascular spaces) was assessed on 3T magnetic resonance imaging. Multivariate analyses examined association pathways among these variables.

**RESULTS:**

Neighborhood deprivation was associated with poorer cognition (*r* = 0.36, *p* < 0.001), greater prevalence of modifiable risk factors (*r* = 0.36, *p* < 0.001), and greater SVD burden (*β* = 0.18, *p* = 0.008). Serial mediation showed that the effects of deprivation on cognition were indirect, possibly operating via lifestyle risk and SVD, explaining 20% of the total effect, whereas SVD alone explained 28%.

**DISCUSSION:**

Neighborhood disadvantage relates to poorer cognition, possibly mediated through vascular risk factors and cerebrovascular disease.

**Highlights:**

- Neighborhood deprivation linked to poorer cognition in healthy midlife adults
- Deprivation linked to small vessel disease (SVD) and modifiable risk factors (chiefly cardiovascular risk)
- Association between deprivation and cognition mediated by modifiable risk and SVD
- Mediation was exclusive to hypertensive SVD, but not cerebral amyloid angiopathy (CAA)‐related SVD

## Graphical abstract

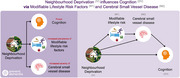

## 1 INTRODUCTION

Dementia is a global problem that disproportionately affects socioeconomically disadvantaged populations. This disparity is evident at both the global and local levels, with recent reductions in dementia incidence occurring predominantly in high‐income countries and more affluent areas within countries.^1^, ^2^ It is important to note that although an estimated 45% of dementia cases may be preventable by addressing modifiable risk factors, the greatest potential for impact lies in low‐ and middle‐income countries (LMICs) and socioeconomically disadvantaged groups, where risks are more prevalent and less well‐managed.^1^, ^3^

Within countries, individuals living in disadvantaged neighborhoods show greater cognitive decline ^4^, ^5^ and higher dementia risk,^6^, ^7^ independent of individual socioeconomic status.^8^ Structural brain changes have also emerged in recent years, with neighborhood deprivation being linked to smaller hippocampal volume^9^ and cortical thinning^4^, ^10^ on magnetic resonance imaging (MRI) and greater neuropathology in autopsy samples.^11^ However, mechanisms linking neighborhood deprivation to brain changes and cognition remain unclear, although several have independently alluded to a possibly vascular pathway.^1^, ^10^, ^12^

Understanding the mechanisms linking neighborhood deprivation and dementia represents a pressing area of research to guide population‐level dementia prevention efforts. There is a robust body of evidence that residents of disadvantaged areas typically have lower access to healthy food options, increased stressors, fewer recreational opportunities, and a myriad of structural factors that could influence risky health behaviors and cardiovascular health.^13^, ^14^, ^15^, ^16^, ^17^ Considering the growing recognition that addressing modifiable risk factors like obesity and hypertension could mitigate dementia by reducing vascular damage,^1^, ^9^, ^12^ understanding these mechanisms could have far‐reaching implications on dementia prevention.^18^, ^19^

Given prior evidence of the significance of modifiable dementia risk factors and cerebral small vessel disease (SVD) as early as midlife,^20^, ^21^, ^22^ we probed the questions of (1) whether neighborhood deprivation relates to lifestyle risk factors and cognition, and (2) whether these links can be explained by SVD. Using multivariate approaches, we examined both broad (construct‐level) associations and domain‐specific contributions that could guide targeted prevention strategies.

## 2 METHODS

### 2.1 Participants

Participants were cognitively healthy middle‐aged adults (ages 40–59) recruited as part of the PREVENT‐Dementia program; study protocol is detailed elsewhere and in Supporting Information.^21^, ^23^, ^24^, ^25^ Of 700 participants recruited across the United Kingdom and Ireland, 634 had valid postcode data, of which 604 underwent brain MRI. Two participants were excluded due to failed MRI quality checks and 17 were excluded due to incidental MRI findings or changes that precluded imaging analysis (e.g., tumor resection, meningioma), resulting in a final sample size of 585.

### 2.2 Quantification of cerebral SVD

Imaging markers of SVD were assessed on 3T MRI (Siemens; acquisition parameters in Supporting Information) according to the Standards for Reporting Vascular Changes on Neuroimaging (STRIVE) guidelines.^26^ White matter hyperintensity (WMH) volumes were extracted from lesion maps created on fluid‐attenuated inversion recovery (FLAIR) MRI.^27^ All WMH maps were visually inspected and manually corrected for misclassifications, and WMH volumes were normalized by total intracranial volume. WMHs were also visually rated using the Fazekas scale for the computation of composite SVD scores.^28^ Cerebral microbleeds (CMBs) were assessed on 3T susceptibility‐weighted imaging (SWI) scans using the Microbleed Anatomical Rating Scale (MARS),^29^ and cross‐validated on T1‐ and T2‐weighted images to exclude CMB “mimics” (e.g., melanoma). Where uncertain, CMBs were labeled as “*possible* CMB”—this includes scenarios whereby CMBs cannot be distinguished from vascular flow voids. Such cases of “possible CMB” were excluded from analysis, and only “definite CMB” was analyzed. Lacunes were evaluated on T1‐weighted, T2‐weighted, and FLAIR images.^30^ Lacunes and CMBs were classified by location as deep or lobar. Lobar regions were defined according to Stark and Bradley,^31^ comprising cortical and subcortical regions, whereas deep regions included the basal ganglia, thalamus, internal capsule, external capsule, corpus callosum, and deep and periventricular white matter.^29^, ^32^ Perivascular spaces (PVS) were assessed separately in the basal ganglia (BG) and centrum semiovale (CSO) on T2‐weighted scans using a validated rating scale.^33^ Details on SVD quantification and inter‐rater reliability are presented in Supporting Information and previous publications.^20^, ^21^

**RESEARCH IN CONTEXT**

1. **Systematic review**: A PubMed search identified studies (predominantly in the United States, and more recently in the United Kingdom and Europe) linking neighborhood deprivation to increased dementia risk. Existing research often attributes this link to the direct effects of air pollution, stress, or limited health care access. However, there has been a notable lack of studies that consider how structural disadvantage limits individuals' ability to modify their risk factors. An additional PubMed search did not identify any studies on neighborhood deprivation and cerebral small vessel disease (SVD).
2. **Interpretation**: Our findings suggest that neighborhood deprivation contributes to midlife cognitive impairment through a vascular pathway involving modifiable risk factors and greater SVD burden. This pathway remained significant even after accounting for individual socioeconomic status, highlighting the importance of structural barriers and cerebrovascular health.
3. **Future directions**: Future studies should replicate this work in more diverse samples, and in different countries and cultures, to identify structural barriers to health behaviors and vascular brain health for dementia prevention, ideally through longitudinal studies.

### 2.3 Measures of Neighborhood Deprivation

Neighborhood deprivation was assessed by mapping postcode data to national indices of deprivation. In England, postcodes were mapped to the 2019 Indices of Deprivation,^34^ which measures deprivation across multiple domains at the small‐area level using Lower Layer Super Output Areas (LSOAs), which provide consistently sized statistical units averaging 1500 residents or 650 households. Seven deprivation domains are measured separately: (1) *Income Deprivation* measures the proportion of the population experiencing deprivation relating to low income, including individuals who are out of work; (2) *Employment Deprivation* is based on the proportion of the working age population who are involuntarily excluded from the labor market, for example, owing to unemployment, disability, and caring responsibilities; (3) *Education, Skills, and Training Deprivation* measures the lack of attainment and skills in the local population; (4) *Health Deprivation and Disability* measures the risk of premature death, disability, and impairment of quality of life through poor physical or mental health; (5) the *Crime* domain measures the risk of personal and material victimization (violence, burglary, theft, criminal damage); (6) *Barriers to Housing and Services* measures two forms of barriers: *Geographical Barriers*, which relate to the physical proximity of local services (e.g., post office, supermarket, general practitioner (primary care) clinic), and *W* *ider* *B* *arriers*, which pertain to accessibility to housing (e.g., homelessness, household overcrowding, housing affordability); (7) *Living Environment* indicates the quality of the local environment, which includes both the indoor quality of housing (e.g., proportion of houses in poor condition, or without central heating) and the quality of the outdoor living environment (air quality, road accidents). Overall deprivation indices were also extracted for participants residing in Scotland and Ireland using the Scottish IMD 2020 and Pobal HP Deprivation Index 2022, which were combined with the English dataset (further details in Supporting Information). Domain‐level data were not combined, given the differences between the domains across the three countries.^34^, ^35^, ^36^ Deprivation rankings were reverse‐coded to aid interpretability, such that higher deprivation ranks indicate greater deprivation in all measures.

### 2.4 Clinical and neuropsychological assessment

Modifiable lifestyle risk factors were assessed with 13 risk factors, including 11 of the 14 risk factors from the *2024 Lancet Commission on dementia prevention, intervention, and care* proposed to account for up to 45% of dementia risk.^1^ Selection of risk factors is explained under Supporting Information. Depression was assessed with the Center for Epidemiologic Studies Depression Scale (CES‐D).^37^ Traumatic brain injury (TBI) was assessed using the Brain Injury Screening Questionnaire (BISQ)^38^ and analyzed as a continuous variable, that is, number of TBI events. Physical inactivity and social isolation were assessed using the Lifetime of Experiences Questionnaire. Fasting glucose levels were analyzed as a marker of diabetes. Sleep quality was measured using the Pittsburgh Sleep Quality Index (PSQI).^39^ Alcohol intake was a continuous variable of units per week, whereas smoking was measured in cigarettes per week. Diet was evaluated using the Mediterranean Diet Score (Pyramid) derived from the Scottish Collaborative Group Food Frequency Questionnaire; to avoid double counting, alcohol was removed from the total calculation of the Pyramid score.^40^ Systolic blood pressure was averaged from three readings. Low‐density lipoprotein (LDL) cholesterol was analyzed as a continuous variable. Obesity was measured as a continuous measure of waist‐to‐hip ratio. Hearing impairment was a self‐reported binary variable. Cognition was assessed using the computerized COGNITO battery.^41^ Composite cognitive domain scores were computed by averaging the *z*‐scores of relevant tasks in each domain, and reverse coded for interpretability, such that higher scores indicate poorer cognitive performance (further details in Supporting Information).^41^, ^42^

### 2.5 Statistical analysis

Normality was assessed using the Shapiro–Wilk test, with non‐normal variables transformed to address skewness. Two forms of multivariate approaches were adopted. Canonical Correlation Analysis (CCA) identified domain‐specific associations by extracting maximally correlated variable combinations,^43^ whereas Structural Equation Modeling (SEM)^44^, ^45^ was employed for mediation analysis and broader (construct‐level) analysis.

To test the association of neighborhood deprivation and cognition and identify domain‐specific effects, we performed CCA using a two‐level analytical approach.^46^, ^47^, ^48^, ^49^ In first‐level analysis, permutation‐based CCA was conducted to assess the relationship between the two constructs, each represented as separate multivariate datasets. The first dataset represented *Neighborhood Deprivation* and included the seven domains of deprivation; the second dataset represented six cognitive domains (memory, attention, executive function, visuospatial, language, processing speed). Missing data were imputed using default settings in the *multivariate imputation by chained equations* (*mice*) package, and all variables were standardized into *z*‐scores for CCA. Significance of the CCA model was tested via permutation testing with 5000 iterations. In the second‐level analysis, we extracted subject scores for each pair of canonical components to examine whether associations between the constructs remained significant in a simple linear regression analysis adjusting for sex, age, and education (see Supporting Information, Specification of confounders). Canonical weights were examined to determine the contribution of different variables within the canonical variate.

To evaluate the association of neighborhood deprivation with modifiable lifestyle risk, the same CCA approach was performed. The first dataset of neighborhood deprivation remained constant, whereas the second dataset represented *Modifiable Lifestyle Risk* and comprised the 13 lifestyle risk variables described in Section 2.4.

SEM was conducted to examine whether the association between neighborhood deprivation (independent variable; IV) and cognition (dependent variable; DV) was mediated through modifiable lifestyle risk factors (M1) and SVD (M2). Confirmatory factor analysis was first performed to evaluate the measurement quality of the latent variables of SVD (total WMH volume, PVS in basal ganglia, microbleed presence, lacune presence)^50^ and neighborhood deprivation (estimated from the seven domains of deprivation; see Section 2.3). Given the mix of continuous (e.g., WMH) and categorical (e.g., microbleed presence, lacune presence), models were fitted using a robust weighted least squares estimator weighted least squares mean and variance adjusted (WLSMV). Model fit was assessed using standard goodness‐of‐fit indices and cutoffs: Comparative Fit Index (CFI >0.95), Root Mean Square Error of Approximation (RMSEA <0.05), and Standardized Root Mean Square Residual (SRMR <0.05).^51^ Model modification was performed in a systematic stepwise approach by examining the modification indices derived from Lagrange Multiplier tests to identify potential sources of misfit that could improve model fit if addressed. Re‐specifications were considered when modification indices were large (>3.84) and implemented if the proposed changes were theoretically sound.^52^ Confirmatory Factor Analysis demonstrated good model fit for the latent model of SVD (CFI = 1, RMSEA = 0, SRMR = 0.018), but not the latent model of deprivation (CFI = 0.854, RMSEA = 0.216, SRMR = 0.110). As double‐counting is a known issue in the construction of the IMD deprivation index, we examined whether specifying the paths of residual correlations between the seven domains improved model fit adequately. Using a stepwise approach based on modification indices, the latent model of deprivation achieved good fit (CFI = 1, RMSEA = 0, SRMR = 0.013). Next, we modeled the association between neighborhood deprivation and SVD. In the full structural model, regression coefficients were assessed and non‐significant paths were removed. The full structural model between the latent variables and neighborhood deprivation accounted for sex, age, and education, and achieved good model fit (CFI = 1, RMSEA = 0, SRMR = 0.013).

Mediation analysis was then conducted with 5000 bootstrap samples, accounting for the same covariates. Three models were fitted: Model 1 was a linear mediation model with neighborhood deprivation as the exposure, lifestyle and SVD as mediators, and cognition as the outcome. Model 2 extended this by adding non‐linear effects of deprivation using quadratic terms in all mediator and outcome regressions. Model 3 further extended this with the inclusion of exposure–mediator interaction terms (Deprivation*Lifestyle, Deprivation*SVD) to account for potential effect modifications.^53^ To examine the differential effects of SVD subtypes, we repeated this analysis by replacing markers of global SVD (total WMH, PVS‐BG, presence of CMB, presence of lacunes) with hypertensive arteriopathy (deep WMH, PVS‐BG, presence of deep CMB, presence of deep lacunes) and CAA‐type SVD (occipital WMH, PVS‐CSO, presence of lobar CMB, presence of lobar lacunes).[Table alz70756-tbl-0001] All SEM models were over‐identified, and model fit indices (CFI, RMSEA, SRMR) are reported in the corresponding results tables. Several forms of sensitivity analyses were conducted. First, we applied a counterfactual‐based approach in separate mediation models for each mediator.^53^, ^54^ This allowed the decomposition of the total effect into four basic components: Controlled Direct Effect (CDE), Reference Interaction (INT_ref_), Mediated Interaction (INT_med_), and Pure Indirect Effect (PIE), while accounting for potential exposure–mediator interaction effects (further details in Supporting Information).^53^ Next, we examined the robustness of our indirect effects to potential unobserved confounders using the *medsens* function from the *mediation* R package to determine the degree of residual correlation (rho) at which the average causal mediation effect (ACME) would become non‐significant.^55^ Next, we fit two models with lifestyle and SVD as mediators in separate general regression models, adjusting for the same covariates of sex, age, and education. We then additionally examined these pathways assuming independent (rather than sequential) mediating effects of modifiable risk factors and SVD by estimating a parallel mediation model treating lifestyle risk and SVD as independent mediators (Figure S1). Finally, we conducted further sensitivity analyses to assess the robustness of our mediation models to violations of the missing at random (MAR) assumption. We applied a delta‐adjustment approach to missing values imputation, shifting imputed values upwards by 0.5 units to simulate a missing not at random (MNAR) mechanism, under the assumption that individuals with missing data have systematically higher risk profiles. SEM models were then re‐estimated using the MNAR datasets.

Analyses were conducted on R v4.4.0, with statistical significance set at *p = 0*.05 in two‐tailed tests. To reduce collinearity, all independent variables in regression models were standardized to *z*‐scores.

## 3 RESULTS

### 3.1 Cohort characteristics

Our cohort of 585 participants had a median age of 52 (mean 51.1, standard deviation [SD] 5.5), and 61.9% were female (*n* = 362) (Table 1). Greater neighborhood deprivation related to fewer years of education (*r* = 0.14, *p <* *0*.001), but did not differ by sex (*t* = 0.67, *p = 0*.505) or age (*r* = 0.42, *p = 0*.671).

**TABLE 1 alz70756-tbl-0001:** Sample characteristics.

	England	Scotland	Ireland	OVERALL
*N*	331	183	71	**585**
Sex, *N* (%)				
*Female*	230 (69.5%)	102 (55.7%)	30 (42.3%)	**362 (61.9%)**
*Male*	101 (30.5%)	81 (44.3%)	41 (57.7%)	**223 (38.1%)**
Age (in years), mean ± SD	51.3 ± 5.4	51.2 ± 5.6	50.1 ± 5.8	**51.1 ± 5.5**
Education (in years), mean ± SD	16.6 ± 3.3	16.7 ± 3.1	17.4 ± 3.4	**16.7 ± 3.2**
APOE4, *N* (%)	116 (35.4%)	70 (38.7%)	29 (41.4%)	**215 (37.1%)**
Family history of dementia, *N* (%)	178 (54.1%)	88 (48.6%)	40 (56.3%)	**306 (52.7%)**
Hypertension, *N* (%)	56 (16.9%)	50 (27.5%)	19 (26.8%)	**125 (21.4%)**
Hyperlipidaemia, *N* (%)	62 (18.7%)	37 (21.8%)	16 (23.9%)	**115 (20.2%)**
Diabetes mellitus, *N* (%)	6 (1.8%)	7 (4.2%)	3 (4.2%)	**16 (2.8%)**
Obese, *N* (%)	72 (21.7%)	64 (35.4%)	17 (23.9%)	**153 (26.2%)**
Traumatic brain injury, *N* (%)	124 (37.6%)	60 (32.8%)	27 (38%)	**211 (36.1%)**
Current smoker, *N* (%)	17 (5.1%)	8 (4.4%)	9 (12.7%)	**34 (5.8%)**
High alcohol intake, *N* (%)^a^	45 (13.6%)	39 (21.4%)	1 (1.4%)	**85 (14.6%)**

Abbreviations: APOE4, apolipoprotein epsilon 4; SD, standard deviation

^a^ High alcohol intake is defined as >21 units per week.

### 3.2 Neighborhood deprivation and cognition

**FIGURE 1:**
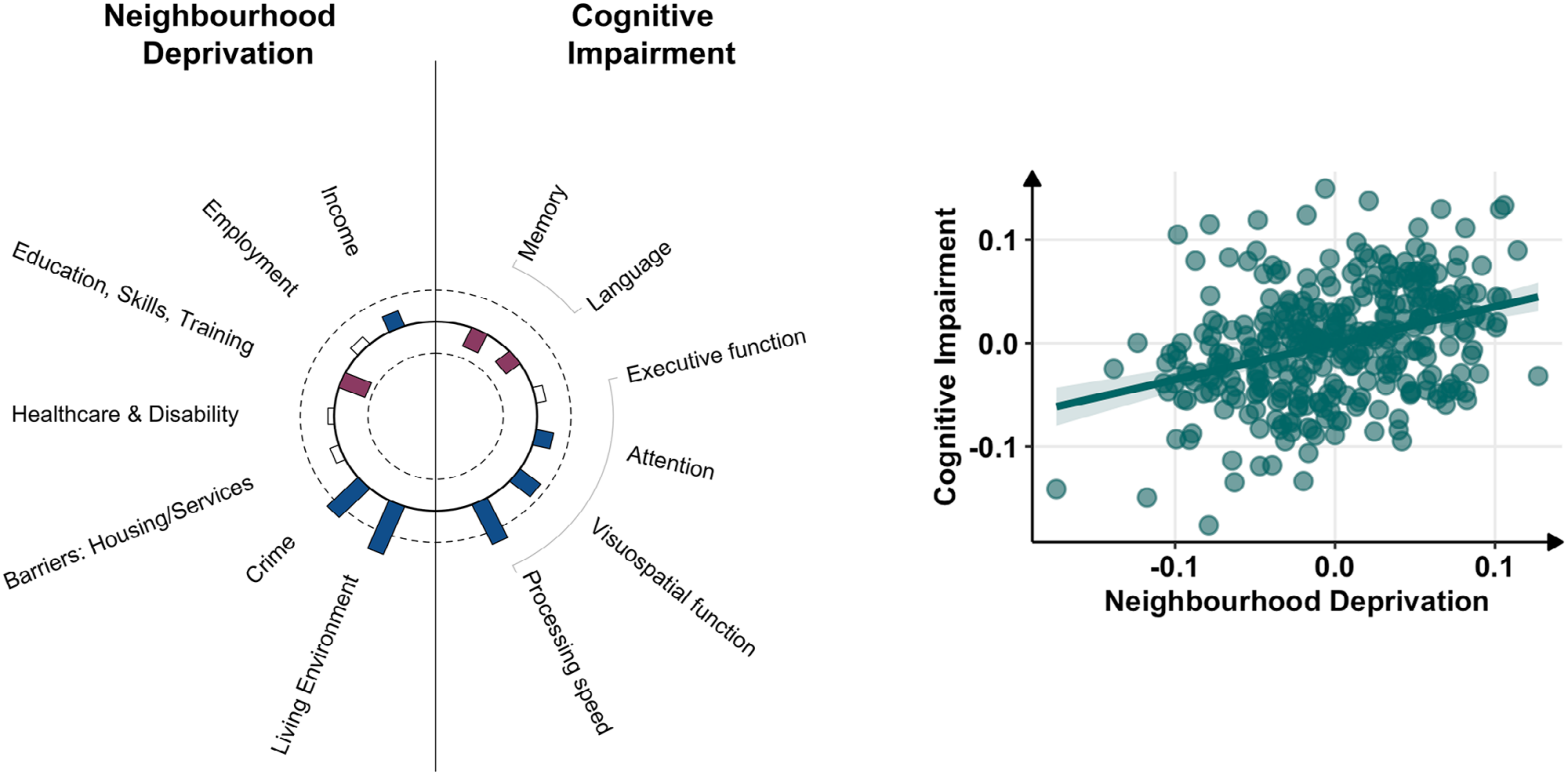
Canonical correlation analysis between neighborhood deprivation and cognition. Left: Heliograph of canonical variate loadings. Blue bars extending outward represent positive weights (i.e., greater deprivation/greater cognitive deficits); red bars extending inward represent negative weights (i.e., lower deprivation/lower cognitive deficits); uncolored bars indicate |r| < 0.2; length indicates strength of structural correlations (loadings). Half‐maximum strength of correlation is represented by the innermost (*r* = −0.5) and outermost (*r* = 0.5) circles. Right: Bivariate correlation plot of canonical correlations between neighborhood deprivation and cognition (residuals adjusted for sex and age).

CCA demonstrated a significant association between neighborhood deprivation and cognition in one significant component (*r* = 0.36, *p <* *0*.001) (Figure 1). This association remained significant in permutation testing (stat = 0.13, *p <* *0*.001), in adjusted analysis using simple linear regression analysis of composite variate scores adjusting for sex, age, and education (*β* = 0.35, *t* = 6.97, *p <* *0*.001), and sensitivity analysis using linear mixed‐effects modeling of combined deprivation ranks (*β* = 0.14, 95% confidence interval [CI]: 0.04–0.25, *p = 0*.008). Assessment of the canonical variate loadings suggests that greater deprivation in the domains of living environment (0.82), crime (0.64), and income (0.26) were associated with poorer cognitive performance in processing speed (0.69), visuospatial functioning (0.39), and attention (0.26). Exceptions were observed, with reverse weights in the deprivation domain of education (−0.42) and the cognitive domains of memory (−0.32) and language (−0.27). Table S1 provides the full list of canonical variate loadings.

### 3.3 Neighborhood deprivation and modifiable risk factors

**FIGURE 2:**
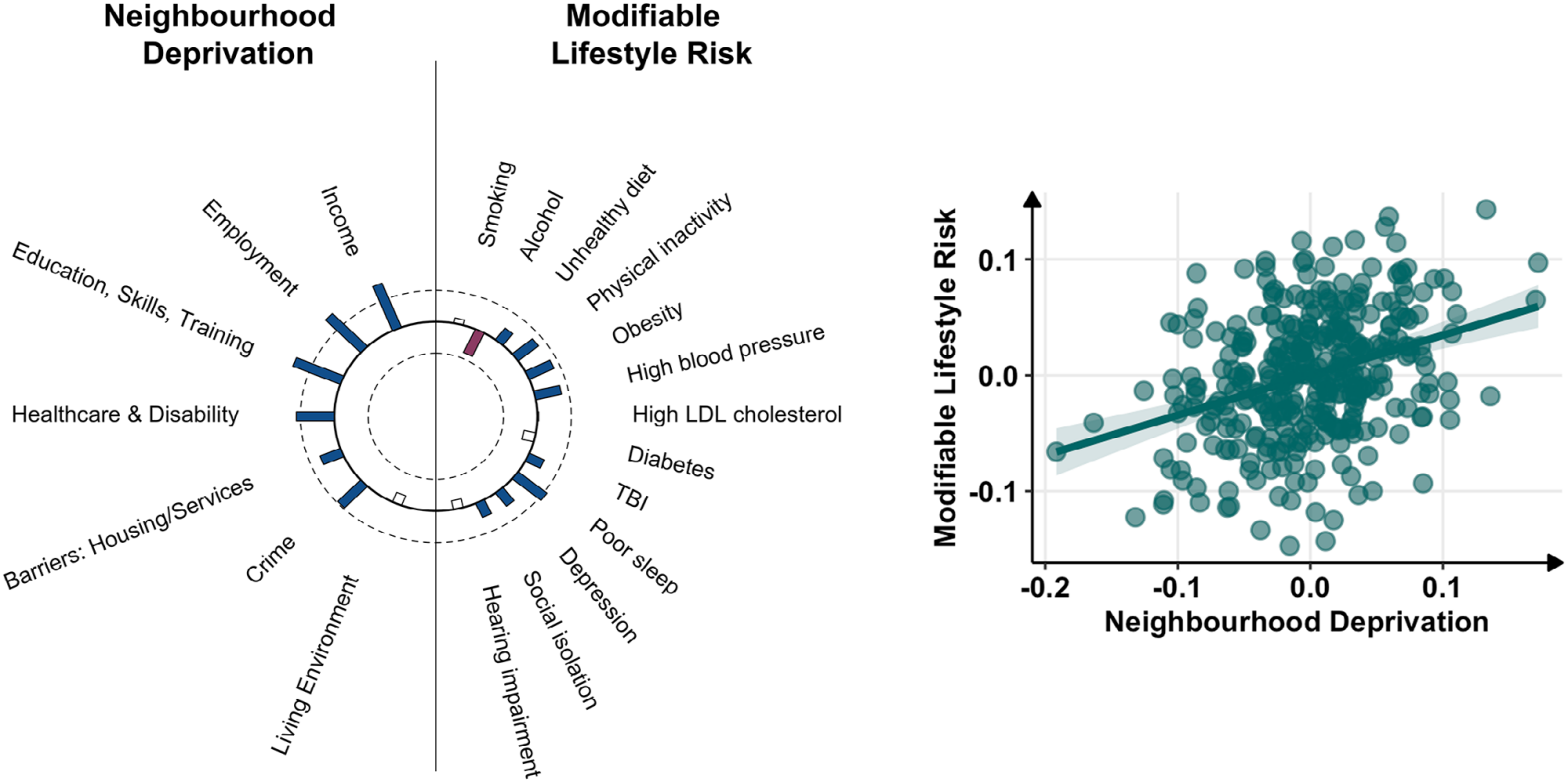
Canonical correlation analysis between neighborhood deprivation and modifiable lifestyle risk. Left: Heliograph of canonical variate loadings. Blue bars extending outward represent positive weights (i.e., greater deprivation/greater lifestyle risk); red bars extending inward represent negative weights (i.e., lower deprivation/ lower lifestyle risk); uncolored bars indicate |r| < 0.2; length indicates strength of structural correlations (loadings). Half‐maximum strength of correlation is indicated by the innermost (*r* = −0.5) and outermost (r = 0.5) circles. Right: Bivariate correlation plot of canonical correlations (residuals adjusted for sex and age). LDL, low‐density lipoprotein; TBI, traumatic brain injury.

CCA identified one significant canonical variate, indicating a significant correlation between neighborhood deprivation and modifiable lifestyle risk factors (*r* = 0.36, *p <* *0*.001), which remained significant with permutation testing based on 5000 permutations (stat = 0.13, *p = *0.016) (Figure 2). This association appeared to be driven by deprivation in the domains of employment (0.74), income (0.72), and education, skills and training (0.71), predominantly affecting the lifestyle risk factors of poor sleep (0.48), physical inactivity (0.42), obesity (0.40), and high blood pressure (0.38). Notably, alcohol intake was weighted in the opposite direction (−0.47), such that lower neighborhood deprivation related to greater alcohol consumption. Table S2 presents the full list of canonical variate loadings. Adjusting for sex, age, and education, the association between the canonical component scores of deprivation and modifiable lifestyle risk remained significant (*β* = 0.33, *t* = 6.39, *p <* *0*.001).

### 3.4 Mediation analysis of pathways between neighborhood deprivation and cognition

Accounting for sex, age, and education, SEM demonstrated significant associations between the latent variables of SVD and neighborhood deprivation (*β* = 0.18, est = 0.12, 95% CI: 0.03–0.20, *p = 0*.008) (Figure S2 and Table S3). This was replicated in sensitivity analysis with data imputed under a MNAR assumption (*β* = 0.17, est = 0.11, 95% CI: 0.02–0.20, *p = 0*.015), and sensitivity analysis using linear mixed effects modeling of deprivation rankings (*β* = 0.14, 95% CI: 0.04–0.25, *p = 0*.008).

**FIGURE 3:**
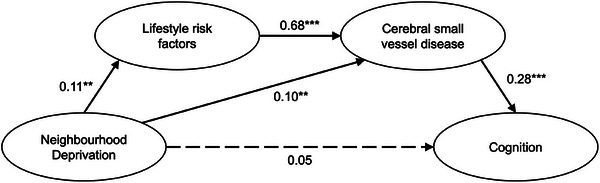
Structural equation modeling of mediation analysis. Values presented are standardized β coefficients. Solid lines indicate statistical significance, whereas dashed lines indicate non‐significant paths. **p < 0.01, ***p < 0.001.

Serial mediation modeling in SEM demonstrated significant mediation (indirect) effects via modifiable lifestyle risk factors and SVD burden (*β* = 0.02, 95% CI: 0.00–0.04), which accounted for 20% of the total effect, that is, proportion indirect contribution (PIC) = 0.20. Mediation also occurred via SVD burden alone (*β* = 0.03, 95% CI: 0.01–0.05), which accounted for 28% of the total effect (PIC = 0.28) (Figure 3, Table 2). The direct effect of neighborhood deprivation on cognition was non‐significant, indicating a fully mediated pathway via Lifestyle and SVD. Findings remained consistent in sensitivity analyses including quadratic and exposure–mediator interaction terms in the model. Full parameter estimates and model fit indices for all three models are presented in Table 2. We repeated these analyses, replacing global SVD with measures of two SVD subtypes—CAA‐SVD and hypertensive arteriopathy (HA‐SVD). Consistent with the original model, the HA‐SVD model exhibited full mediation, as both indirect effects were significant, whereas the direct effect was non‐significant (Table S4). Similarly, in the CAA‐SVD model, the indirect effect via Lifestyle and CAA‐SVD was significant (*β* = 0.04, 95% CI: 0.01–0.07; Table S5), suggesting the presence of a mediating pathway via Lifestyle and CAA‐SVD combined. However, the indirect effect via CAA‐SVD alone was non‐significant (*β* = 0.02, 95% CI: −0.01 to 0.05), suggesting that neighborhood deprivation does not influence cognition through CAA‐SVD alone, without lifestyle risk factors playing a role. Sensitivity analysis accounting for nonlinearity and interactions in the HA‐SVD and CAA‐SVD models can be found in Table S4 and Table S5, respectively.

**TABLE 2:** Serial mediation analysis path coefficients and model fit indices.

	Model 1	Model 2	Model 3
**Direct Effects**			
Deprivation → Lifestyle	0.11 [0.02, 0.19]	0.10 [0.02, 0.18]	0.10 [0.02, 0.18]
Deprivation → Lifestyle (Quadratic)	NA	0.03 [−0.06, 0.11]	0.03 [−0.06, 0.11]
Deprivation → SVD	0.10 [0.03, 0.18]	0.10 [0.02, 0.18]	0.10 [0.02, 0.18]
Deprivation → SVD (Quadratic)	NA	0.01 [−0.07, 0.09]	0.01 [−0.07, 0.09]
Lifestyle → SVD	0.68 [0.58, 0.78]	0.68 [0.58, 0.78]	0.68 [0.58, 0.78]
SVD → Cognition	0.28 [0.17, 0.39]	0.17 [0.03, 0.29]	0.18 [0.04, 0.30]
Deprivation → Cognition	0.05 [−0.04, 0.15]	0.05 [−0.04, 0.15]	0.05 [−0.04, 0.15]
Deprivation → Cognition (Quadratic)	NA	0.09 [0.00, 0.18]	0.11 [0.01, 0.20]
Deprivation × Lifestyle → Cognition	NA	NA	−0.07 [−0.23, 0.07]
Deprivation × SVD → Cognition	NA	NA	0.02 [−0.12, 0.15]
**Indirect Effects**			
Deprivation → Lifestyle → SVD → Cognition	0.02 [0.00, 0.04]	0.01 [0.00, 0.03]	0.02 [0.00, 0.04]
Deprivation → SVD → Cognition	0.03 [0.01, 0.05]	0.02 [0.00, 0.04]	0.01 [0.00, 0.03]
**Total Effect**	0.10 [0.00,0.20]	0.19 [0.06, 0.33]	0.15 [−0.05, 0.32]
**Model Fit**			
CFI	0.988	0.999	0.948
RMSEA	0.062	0.015	0.089
SRMR	0.026	0.019	0.042

*Note*: Model 1: Linear structural equation model. Model 2: Non‐linear structural equation model. Model 3: Non‐linear structural equation model accounting for exposure–mediator interaction. Cognition was reverse coded for consistency across outcome measures, that is, higher scores indicate poorer outcome. Parameter estimates are presented with 95% CI.

Abbreviations: CI, confidence interval; CFI, Comparative Fit Index; RMSEA, Root Mean Square Error of Approximation; SRMR, Standardized Root Mean Square Residual; SVD, cerebral small vessel disease.

We conducted sensitivity analysis using a counterfactual‐based framework to perform a four‐way decomposition of effects accounting for exposure–mediator interactions, which were aligned with our SEM results. This analysis revealed that the total effect of neighborhood deprivation on cognition was driven primarily by *pure indirect effects* via Lifestyle (est = 0.03, 95% CI: 0.00–0.06) and SVD (est = 0.04, 95% CI: 0.01–0.08). *Controlled direct effects* were not statistically significant (Lifestyle: est = 0.07, 95% CI: −0.03 to 0.17; SVD: est = 0.05, 95% CI: –0.05 to 0.15); all estimates and 95% CIs are reported in Table S6. Consistent with Model 3 fitted in SEM, there was no evidence of significant exposure–mediator interaction effects (deprivation*lifestyle: est = −0.02 [−0.12 to 0.08]; deprivation*SVD: est = −0.002 [−0.10 to 0.09]). Next, we assessed the sensitivity of individual mediation pathways of lifestyle and SVD separately using general linear models. The ACME remained positive until the residual correlation between the mediator and outcome (rho) exceeded 0.3 in both the lifestyle‐mediated pathway (rho_ACME(0)_ = 0.3, R^2^_M_ x R^2^_Y_ = 0.09, R^2^_M_ ∼ R^2^_Y_ = 0.045) and the SVD‐mediated pathway (rho_ACME(0)_ = 0.3, R^2^_M_ x R^2^_Y_ = 0.09, R^2^_M_ ∼ R^2^_Y_ = 0.052) (Figure S3). In other words, the ACME would be reduced to zero if an unobserved confounder induces a residual correlation of 0.3 between the mediator and outcome models (rho_ACME(0)_ = 0.3), which corresponds with a scenario where the unobserved confounder explains 9% of the residual variance in both models (R^2^_M_ x R^2^_Y_ = 0.09), or roughly 5% of the residual variance in each model individually [R^2^_M_ ∼ R^2^_Y_ = 0.045 (lifestyle) / 0.052 (SVD)], assuming equal confounding strength in both models. Next, in sensitivity analysis using a parallel mediation model, the mediation effect remained statistically significant (est = 0.05 [0.02–0.09]) (Table S7). However, the model exhibited poor overall fit (CFI = 0.622, RMSEA = 0.346, SRMR = 0.097), and modification indices indicated a strong empirical justification for adding a path from lifestyle to SVD (MI = 143.54), supporting the theoretical rationale for the serial mediation framework. Finally, sensitivity analysis was conducted to re‐estimate SEM mediation models under a MNAR assumption using delta‐adjusted multiple imputation,^56^ which revealed consistent results with those reported under MAR assumptions, such that the indirect effects remained statistically significant in Models 1–3. The magnitude and direction of effects remained stable, with little change compared to the MAR‐based models. Path coefficients of all three models (linear, nonlinear, nonlinear + interactions) and their respective model fit indices are presented in Table S8.

## 4 DISCUSSION

In our cohort of healthy middle‐aged adults, we observed a link between neighborhood deprivation and cognition that was explained by greater prevalence of potentially modifiable lifestyle risk factors and consequent SVD. Using multivariate analysis, we identified distinct components driving these associations, which appeared to be underscored by cardiovascular‐specific lifestyle factors, and cognitive deficit patterns consistent with SVD. Of note, the mediating role of hypertensive SVD (but not CAA‐SVD) further highlighted the vascular pathway linking neighborhood deprivation to cognition.

The link between neighborhood deprivation and modifiable lifestyle risk factors appeared to be driven primarily by the deprivation domains of income, education, and employment, which together may represent human capital and economic factors of the neighborhood. Among the 13 modifiable risk factors analyzed, the association with neighborhood disadvantage was most closely tied to lifestyle factors with known cardiovascular underpinnings such as poor sleep, obesity, physical inactivity, and high blood pressure. Socioeconomically deprived areas may perpetuate such risks through limited access to healthy food options and safe recreational spaces, stemming from both individual and contextual constraints.^16^ Most risk factors were more prevalent in disadvantaged neighborhoods, with the notable exception of alcohol consumption, which was lower. This lower rate of alcohol consumption may stem from reduced access to outlets that sell or serve alcohol (e.g., supermarkets, pubs), or limited financial resources that make purchasing non‐essential items like alcohol less feasible. Conversely, individuals living in more affluent areas may have greater access to alcohol through a higher density of outlets, better transport options, and greater spending power—this may be compounded by greater exposure to alcohol use in both social and professional settings and as a coping mechanism for high‐stress occupations.^57^ Our findings are aligned with past studies, which additionally highlight a crucial caveat—that despite consuming less alcohol on average, individuals with lower socioeconomic status face a disproportionately higher risk of alcohol‐related health problems.^58^, ^59^

Individuals living in more deprived neighborhoods exhibit lower cognitive scores. This was driven by the deprivation domains of crime and living environment. Relative to other domains, these pertain to external factors more than internal. Specifically, the crime domain reflects the broader social environment, affecting all residents regardless of individual circumstances. Similarly, Living Environment captures housing quality (e.g., heating, building disrepair) and the surrounding outdoor environment (e.g., green spaces). Together, these could represent daily stressors to residents concerned about physical and social safety. In terms of cognition, the domains most closely linked to neighborhood deprivation were processing speed, visuospatial function, and attention—notably, these cognitive functions are well‐known to be affected in SVD, and represent the earliest deficits in vascular cognitive impairment.^60^ Of interest, inverse weightings were observed in the Education deprivation domain, and the memory and language domains of cognition, which may suggest the presence of interaction effects between specific variables, or a domain‐specific effect of education on memory and language, which has been reported previously.^61^

The association between neighborhood deprivation and cognition was mediated by an increased prevalence of lifestyle risk factors and consequently poorer cerebrovascular health. It is notable that this pathway differed between SVD subtypes. On top of global SVD, we also studied the two main subtypes of SVD—hypertensive SVD (HA‐SVD) and CAA‐SVD: HA‐SVD is closely associated with vascular factors like hypertension, endothelial dysfunction, and blood–brain barrier dysfunction, whereas CAA‐SVD reflects vessel damage caused by amyloid protein accumulation within vessel walls.^62^, ^63^ The full serial mediation pathway (*Deprivation → Lifestyle → SVD → Cognition*) was significant across all three SVD models. Of interest, the alternative pathway without lifestyle risk factors (*Deprivation → SVD → Cognition*) was also significant. The significant association between neighborhood and SVD—independent of lifestyle factors—may reflect the impact of structural and environmental factors on cerebrovascular health directly, not as a function of lifestyle. For instance, chronic exposure to psychosocial stress (e.g., stemming from perceived lack of safety as discussed above) has been linked to changes in the cerebrovasculature such as blood–brain barrier dysfunction and neuroinflammation, which are key mechanisms implicated in SVD.^64^, ^65^ These adverse effects on vascular health may also be associated with greater exposure to air pollution, noise pollution, and poorer living conditions experienced by residents of disadvantaged neighborhoods. Notably, this alternative pathway without lifestyle risk factors (*Deprivation → SVD → Cognition*) was significant only in the global SVD and hypertensive SVD models, but not in the CAA‐SVD model. This suggests that neighborhood deprivation does not influence cognition through CAA‐SVD alone, without the involvement of lifestyle risk factors. This exclusive mediation by hypertensive SVD (but not CAA‐SVD) provides further support for the role of cerebrovascular health in the explanatory pathway between neighborhood disadvantage and dementia risk.

This study highlights the role of neighborhood deprivation on midlife cognition, by influencing both behavioral and vascular pathways. Given that structural disadvantage contributes to SVD and poorer cognitive performance, our results underscore the need for interventions that go beyond individual risk modification and address broader upstream social determinants of brain health. By analyzing the specific challenges associated with different neighborhood profiles, we may identify distinct priorities that vary across regions. In wealthier areas, strategies could focus on reducing alcohol consumption. Conversely, initiatives in lower‐income neighborhoods might involve targeted campaigns promoting healthy lifestyles, such as balanced diets, regular exercise, and sufficient sleep. Such campaigns should also aim to eliminate barriers to adopting healthy behaviors, including improving access to affordable healthcare and healthy food options, reducing crime, and providing safe recreational areas for exercise.

### 4.1 Limitations and future directions

The use of cross‐sectional data represents a key limitation, precluding empirical testing of temporal ordering and directionality. In the absence of longitudinal data, our findings should be interpreted as being consistent with—but not conclusive evidence of—a causal mechanistic pathway. Therefore, future longitudinal studies are required to validate the hypothesized mediation pathways proposed. Additional limitations pertain to our sample composition of predominantly White and higher‐educated individuals.^25^ As noted by VanderWeele and Robinson (2014), race‐related variables can confound both the exposure and mediator pathways. Therefore, structural factors influencing these pathways within more diverse populations could be obscured in our ethnically homogenous sample.^66^ Furthermore, although sensitivity analysis demonstrated moderate robustness of the indirect effects of modifiable lifestyle risk and SVD, the influence of unmeasured confounding factors of a moderate residual strength could change the conclusion of our study. This underscores the importance of replication in well‐characterized, diverse cohorts and the need for further research to identify potential unmeasured confounders, as well as alternative explanatory pathways. Other possible sources of biases could stem from the study's recruitment strategy (e.g., recruitment of family members of clinic patients) and platforms (e.g., Join Dementia Research website), which may introduce selection bias, overrepresenting individuals with higher socioeconomic status or those more engaged in health care. Although these may be partially mitigated by using the transformed IMD domain scores, which ensures greater variance in deprivation scores in the most deprived areas, analyses should be replicated in more diverse samples, and in different countries and cultures. To that end, the PREVENT Next Generation study (an extension of the PREVENT‐Study) has prioritized inclusive and diverse recruitment, including racialized, neurodiverse, and lower socioeconomic groups.^67^

To date, research on neighborhood deprivation has been conducted predominantly in the United States,^4^, ^6^, ^9^, ^11^, ^68^ with growing evidence emerging more recently from the United Kingdom ^5^, ^12^ and Europe.^8^ Notably, such research is still lacking in LMICs, where cultural and systemic influences of social and environmental factors could differ substantially, potentially identifying priorities and targets for dementia prevention that differ from higher‐income countries.^68^, ^69^ Furthermore, the availability of publicly available indices of deprivation would be especially important in LMICs, given the disproportionately greater potential for dementia risk modification and prevention.^69^ Finally, cultural differences (e.g., attitudes towards alcohol) warrant replication of this finding beyond the United Kingdom.

### 4.2 Conclusion

Our findings highlight an association between neighborhood disadvantage and poorer cognitive performance in otherwise healthy middle‐aged adults, and provide support that this may operate via cardiovascular and cerebrovascular pathways. Although causal inference cannot be definitively established given our cross‐sectional investigation, results are consistent with our hypothesis that neighborhood disadvantage could pose as a contextual hindrance to cardiovascular risk management, thereby increasing cerebrovascular disease burden and, consequently, affecting cognition. Our findings highlight the need for longitudinal studies, suggesting that targeting upstream structural barriers may hold promise for mitigating vascular contributions to cognitive decline, and emphasizing systemic equity over individual responsibility for dementia prevention.

## CONFLICT OF INTEREST STATEMENT

No disclosures were reported. Unrelated to this work, J.T.O'B. has received honoraria for work as data and safety monitoring board (DSMB) chair or member for TauRx, Axon, Eisai, has acted as a consultant for Lilly and Biogen, and has received honorarium for talks from GE Healthcare and research support from Alliance Medical and Merck. Author disclosures are available in the Supporting Information.

## CONSENT STATEMENT

The study was approved by the London‐Camberwell St Giles National Health Service Ethics Committee (REC reference: 12/LO/1023), which operates according to the Helsinki Declaration of 1975 (and as revised in 1983). All participants provided written informed consent.

## Supporting information



Supporting Information

Supporting Information
