# Supplementary material for: Neighborhood deprivation and midlife cognition: Evidence of a modifiable vascular pathway involving health behaviors and cerebral small vessel disease
Source: Alzheimers Dement. 2025 Nov 5;21(11):e70756. doi: 10.1002/alz.70756 (PMC12587304; doi:10.1002/alz.70756)
Supplement: Supplementary file 1 — Supporting Information [file ALZ-21-e70756-s002.docx]

**Neighbourhood deprivation and midlife cognition: evidence of a modifiable vascular pathway involving health behaviours and SVD**

Supplementary Materials

**Recruitment**

Participants were eligible for inclusion in the PREVENT-Dementia Programme if they were aged 40 to 59 years at the time of consent, did not have a diagnosis of dementia or other major neurological disorders, and had no known contraindications to undergoing an MRI scan. Participants were recruited in five sites across the UK and Ireland - West London, Edinburgh, Cambridge, Oxford and Dublin. Recruitment was conducted through a variety of channels across each participating sites with a recruitment strategy targeting a 50-50 balance of individuals with and without a family history of dementia. Initially, participants were recruited through family members of patients attending National Health Service (NHS) memory clinics at the study sites and via local dementia research registers. Subsequent recruitment occurred through word of mouth, with family and friends of enrolled participants invited to join. Participants were recruited via the Join Dementia Research website (<https://www.joindementiaresearch.nihr.ac.uk/>), and interested individuals could register their interest via the PREVENT-Dementia website (<https://preventdementia.co.uk/>) and public presentations and engagement sessions. Recruitment was not geographically restricted, allowing participants to attend any site, provided they were able to travel and complete all major assessments.

**MRI acquisition**

All participants were scanned on 3T Siemens scanners. Three-dimensional T1-weighted (T1w) MPRAGE parameters were as follows: 160 slices, repetition time (TR)=2300ms, echo time (TE)=2.98ms, flip angle=9°, voxel size=1 × 1 × 1mm^3^. T2-weighted (T2w) parameters were as follows: 32 slices, TR=1500ms, TE=80ms, flip angle=150°, voxel size=0.69 × 0.69 × 4mm^3^. Susceptibility weighted imaging (SWI) parameters were as follows: 72 slices, TR=28ms, TE=20ms, flip angle=15°, voxel size=0.72 × 0.72 × 1.2mm^3^. Fluid-attenuated inversion recovery (FLAIR) parameters were as follows: 27 slices, TR=9000ms, TE=94ms, flip angle=150°, voxel size=0.43 × 0.43 × 4mm^3^.

**Quantification of cerebral small vessel disease (SVD)**

For CMB detection, improved sensitivity was afforded by the acquisition of thin-sliced (vs. thick-sliced) 3T (vs. 1.5T) SWI (vs. conventional gradient echo sequences; GRE) scans, which are reported to detect up to triple the number of CMB compared to conventional 1.5T GRE scans,^1^ and are thought to represent the true prevalence of CMB with greater accuracy and reliability. Visual ratings of each SVD marker were done by a single rater, and 20% of scans were rated by a second rater. The subset of 20% was derived from a random sampling of all participants stratified by study site, performed by a blinded third party who was not involved in any SVD ratings. Raters were blinded to all clinical information, and inter-rater reliability results (Cohen’s kappa) were as follows: CMB: 0.74, lacunes: 0.92, PVS: 0.90 in centrum semiovale, 0.85 in basal ganglia, WMH: 0.74 for periventricular, and 0.89 for deep.^2^

**Measures of Neighborhood Deprivation**

As measures were independently ranked within Scottish postcodes, rank-order data had to be standardized within each country before being combined. Specifically, each area’s ranking was divided by the total number of small areas reported in their respective countries (England: 32,844; Scotland: 6,976, Ireland: 3,417) and multiplied by 100 to derive a percentile rank for every small area.

**Neuropsychological assessments**

Composite scores for each cognitive domain were computed by averaging the z-scores of relevant COGNITO tasks in each domain.^3,4^ The composite *memory* domain was derived from recall tasks in the COGNITO battery, including tasks of name recall, face recall, story recall, name-face association, and a test of implicit memory. *Language* was measured using tasks of phoneme comprehension, phonemic fluency, and vocabulary. The *attention* domain was made up of visual attention, auditory attention, visual-auditory attention, working memory, visuospatial span, and reaction time. *Visuospatial* ability comprises tasks on geometric form matching, complex figure copy, and matrix reasoning. *Executive function* was measured using the Stroop test, visuospatial logic, concept association, and semantic fluency. Finally, *processing speed* was measured using the response latency on tasks of geometric form matching, phoneme comprehension, and visuospatial logic which were adjusted for simple reaction time. Further details on the COGNITO have been previously described.^3,4^

**Selection of modifiable risk factors**

Selection of risk factors was guided by our research question, therefore focusing on factors that could reduce dementia risk through *behavioural* modifications, for which data were available. Therefore, we added measures of unhealthy diet and poor sleep, while excluding education, air pollution, and untreated vision loss: diet and sleep were added based on prior evidence that neighbourhood deprivation could impact one’s health through physical and environmental characteristics, e.g., access to healthy food, opportunities for recreation, environmental stressors ^5–7^; education was removed due to its nature as an *early-life* modifiable risk factor; air pollution was removed because it is included in the computation of the English Indices of Deprivation itself (under the subdomain of Living Environment); data on vision loss was not collected in PREVENT. Therefore, the list of modifiable risk factors analysed were hearing loss, high low-density lipoprotein (LDL) cholesterol, depression, traumatic brain injury, physical inactivity, diabetes, smoking, hypertension, obesity, high alcohol intake, social isolation, poor sleep, and poor diet.

**Specification of confounders**

All models were adjusted for age, sex, and years of education, based on their well-established roles as potential confounders in studies of brain health and dementia risk. Age is a major non-modifiable risk factor of cerebral small vessel disease (SVD), cardiovascular disease, and cognitive decline.^8–11^ In our models, age may confound the relationships between neighbourhood deprivation and lifestyle risk factors (exposure-mediator pathway), between lifestyle risk factors and SVD (mediator-mediator pathway), and between SVD and cognition (mediator-outcome pathway). Given that age is also associated with cognitive performance directly, it may also confound the total exposure-outcome pathway. Sex is another important confounder which has been linked to differences in dementia prevalence, SVD, lifestyle risk factors, and the trajectory of cognitive decline.^12,13^ For example, sex differences in modifiable risk factors like smoking or alcohol consumption, or vulnerability to postmenopausal hypertension may confound the associations between neighbourhood deprivation and lifestyle risk factors (exposure-mediator pathway), and mediator-outcome pathways (lifestyle and cognition, SVD and cognition). Finally, education represents a core modifiable risk factor of dementia and a widely adopted proxy for individual socioeconomic status.^14,15^ Educational attainment has been linked to cognitive reserve, health behaviours, and cognition, as well as shaping socioeconomic trajectories that influence residential environment, exposure to neighbourhood deprivation, and vulnerability to downstream consequences. Accordingly, education could have confounding effects on the exposure-mediator pathways (neighbourhood deprivation and lifestyle/SVD), the exposure-outcome pathway (neighbourhood deprivation and cognition), and mediator-outcome pathways (lifestyle and cognition, SVD and cognition).

**Sensitivity analysis using counterfactual-based approach**

Using a counterfactual-based approach,^16^ we derived four-way decomposition of the total effect into four basic components accounting for potential exposure-mediator interaction effects: pure indirect effect (PIE; i.e., effect attributed to mediation only), reference interaction (INT_ref_ ; effect attributed to interaction only), mediated interaction (INT_med_ ; effect attributed to mediation and interaction), and controlled direct effect (CDE; effect attributed neither to mediation nor interaction), as per VanderWeele (2014).^16^ Two models were fitted per mediator – a mediator model, and an outcome model. Mediator models regressed mediators (lifestyle/SVD) on neighbourhood deprivation and covariates (sex, age, education). Outcome models regressed cognition on neighbourhood deprivation, our mediators (lifestyle/SVD), and the interaction term (deprivation*lifestyle/SVD), adjusting for the same covariates. Given that counterfactual-based four-way decomposition is not readily extendable to models with multiple mediators, we conducted separate decomposition analyses for each mediator as a more parsimonious and interpretable sensitivity analysis.

**REFERENCES**

1. Nandigam RNK, Viswanathan A, Delgado P, et al. MR imaging detection of cerebral microbleeds: Effect of susceptibility-weighted imaging, section thickness, and field strength. *American Journal of Neuroradiology*. 2009;30(2):338-343. doi:10.3174/ajnr.A1355

2. Low A, Prats-Sedano MA, McKiernan E, et al. Modifiable and non-modifiable risk factors of dementia on midlife cerebral small vessel disease in cognitively healthy middle-aged adults: the PREVENT-Dementia study. *Alzheimers Res Ther*. 2022;14(1):1-13. doi:10.1186/S13195-022-01095-4

3. Ritchie K, de Roquefeuil G, Ritchie CW, et al. COGNITO: Computerized Assessment of Information Processing. *J Psychol Psychother*. 2014;4(2):136. doi:10.4172/2161-0487.1000136

4. Ritchie K, Carrière I, Su L, et al. The midlife cognitive profiles of adults at high risk of late-onset Alzheimer’s disease: The PREVENT study. *Alzheimer’s and Dementia*. 2017;13(10):1089-1097. doi:10.1016/j.jalz.2017.02.008

5. Diez Roux A V., Mair C. Neighborhoods and health. *Ann N Y Acad Sci*. 2010;1186(1):125-145. doi:10.1111/J.1749-6632.2009.05333.X

6. Kim B, Branas CC, Rudolph KE, et al. Neighborhoods and sleep health among adults: A systematic review. *Sleep Health*. 2022;8(3):322-333. doi:10.1016/J.SLEH.2022.03.005

7. Billings ME, Cohen RT, Baldwin CM, et al. Disparities in Sleep Health and Potential Intervention Models: A Focused Review. *Chest*. 2021;159(3):1232. doi:10.1016/J.CHEST.2020.09.249

8. De Silva TM, Faraci FM. Contributions of Aging to Cerebral Small Vessel Disease. *Annu Rev Physiol*. 2020;82:275-295. doi:10.1146/ANNUREV-PHYSIOL-021119-034338

9. Chung CP, Ihara M, Saima H, Chen LK. Targeting cerebral small vessel disease to promote healthy aging: Preserving physical and cognitive functions in the elderly. *Arch Gerontol Geriatr*. 2023;110:104982. doi:10.1016/J.ARCHGER.2023.104982

10. Salthouse T. Consequences of age-related cognitive declines. *Annu Rev Psychol*. 2012;63:201-226. doi:10.1146/ANNUREV-PSYCH-120710-100328

11. Harada CN, Natelson Love MC, Triebel KL. Normal cognitive aging. *Clin Geriatr Med*. 2013;29(4):737-752. doi:10.1016/J.CGER.2013.07.002

12. Jiménez-Sánchez L, Hamilton OKL, Clancy U, et al. Sex Differences in Cerebral Small Vessel Disease: A Systematic Review and Meta-Analysis. *Front Neurol*. 2021;12:1960. doi:10.3389/FNEUR.2021.756887

13. Arenaza-Urquijo EM, Boyle R, Casaletto K, et al. Sex and gender differences in cognitive resilience to aging and Alzheimer’s disease. *Alzheimer’s and Dementia*. 2024;20(8):5695-5719. doi:10.1002/ALZ.13844

14. Livingston G, Huntley J, Liu KY, et al. Dementia prevention, intervention, and care: 2024 report of the Lancet standing Commission. *The Lancet*. 2024;404(10452):572-628. doi:10.1016/S0140-6736(24)01296-0

15. Livingston G, Huntley J, Sommerlad A, et al. Dementia prevention, intervention, and care: 2020 report of the Lancet Commission. *The Lancet*. 2020;396(10248):413-446. doi:10.1016/S0140-6736(20)30367-6

16. Vanderweele TJ. A unification of mediation and interaction: A 4-way decomposition. *Epidemiology*. 2014;25(5):749-761. doi:10.1097/EDE.0000000000000121

**Figure S1. Graphical representation of serial mediation model and parallel mediation model**


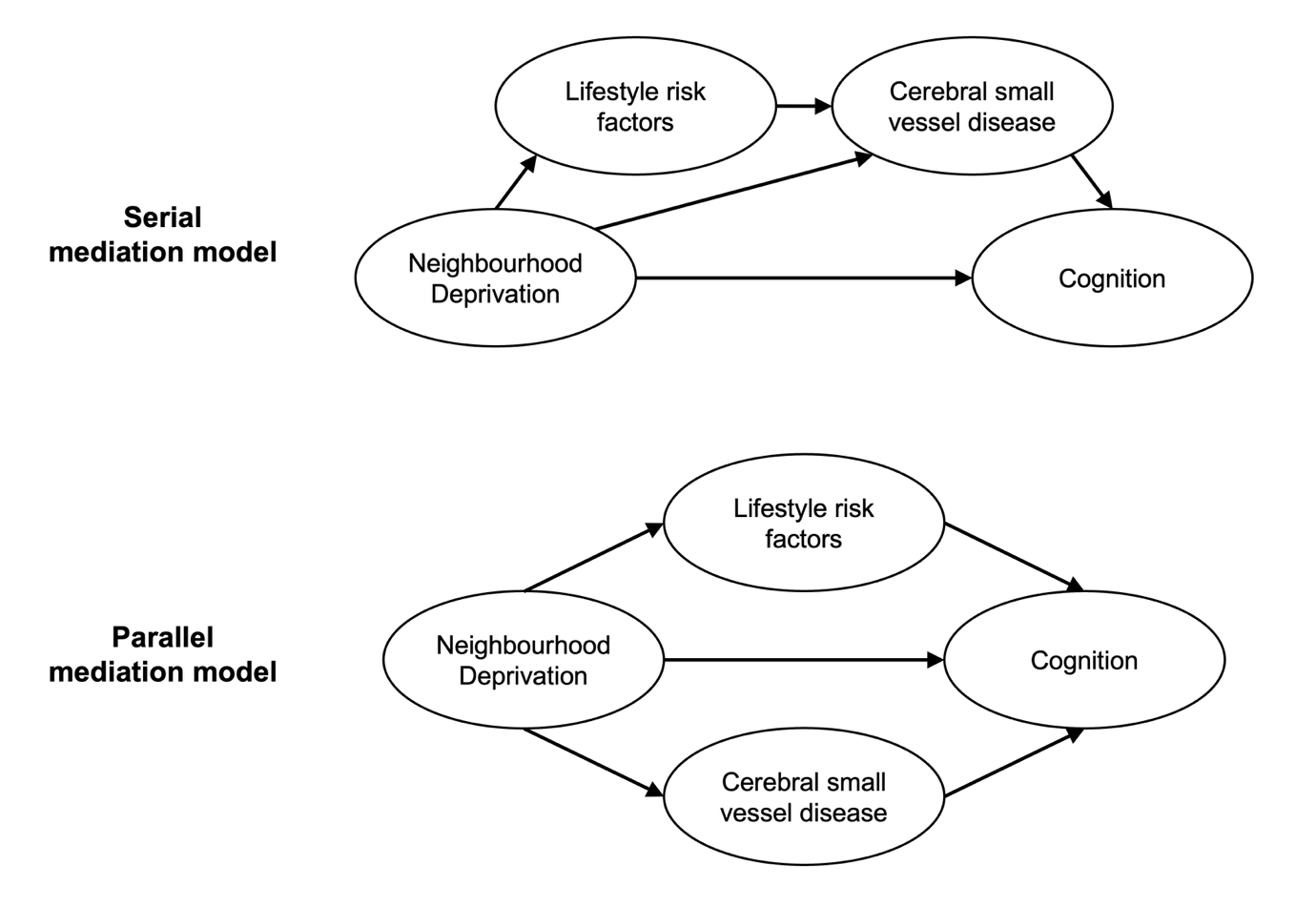


**Figure S2. Structural equation modelling of neighborhood deprivation and cerebral small vessel disease**


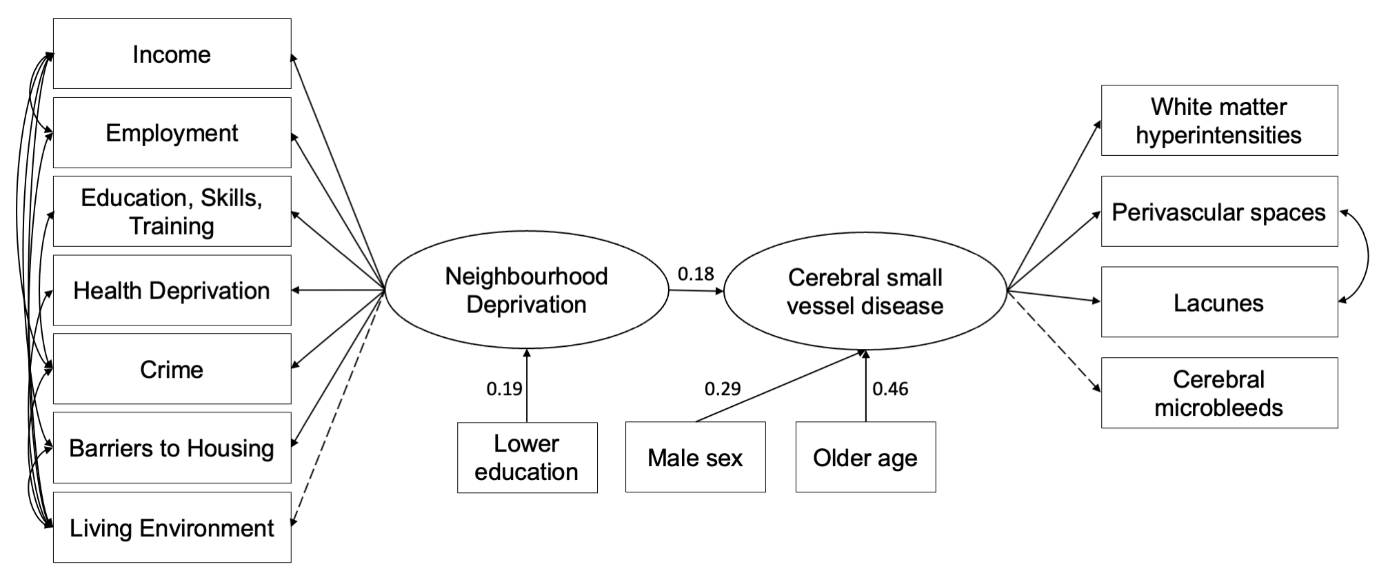


*The full structural model assesses the associations between the latent variables of neighborhood deprivation and cerebral small vessel disease, accounting for years of education, sex, and age. Rectangles represent observed variables; ovals represent latent variables. Values represent standardized beta coefficients. Straight lines represent paths, while double-arrowed curved lines represent covariance. Solid lines indicate statistically significant associations; dashed lines indicate non-significant paths. Path loadings of individual variables can be found in Table S3.*

**Figure S3: Sensitivity analysis to examine robustness towards unmeasured confounding factors.**


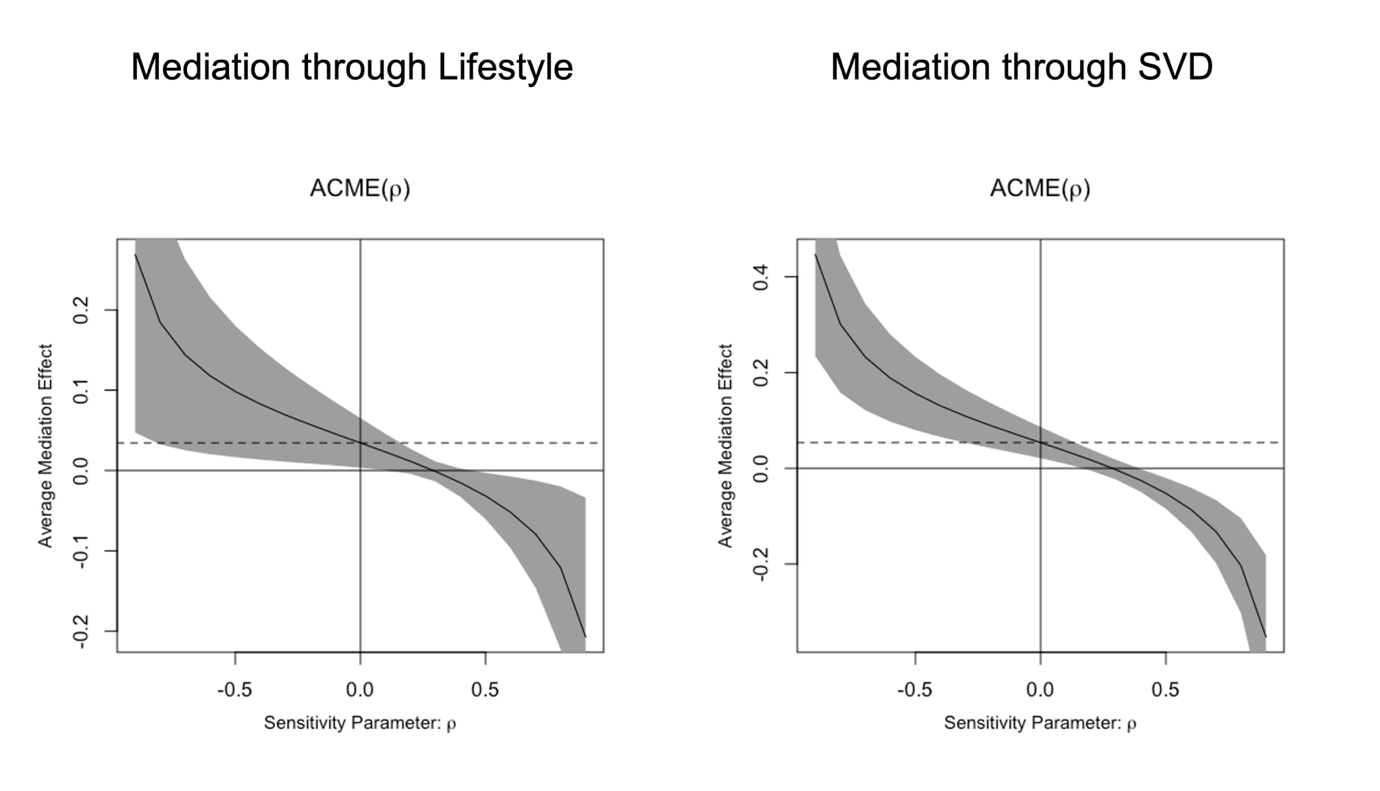


*Plots display the estimated average mediation effect of lifestyle (left) and SVD (right). Dashed lines represent the estimated effect when rho = 0. Solid lines represent the estimated average mediation effect at different levels of rho.*

**Table S1. Canonical correlation analysis between neighbourhood deprivation and cognition.**

|  | **Canonical loadings** |
| --- | --- |
| **Deprivation domains** |  |
| Income | 0.26 |
| Employment | 0.15 |
| Education Skills and Training | -0.42 |
| Health Deprivation and Disability | 0.09 |
| Barriers to Housing and Services | 0.14 |
| Crime | 0.64 |
| Living Environment | 0.82 |
| **Cognitive domains** |  |
| Memory | -0.32 |
| Language | -0.27 |
| Executive function | 0.14 |
| Attention | 0.26 |
| Visuospatial ability | 0.39 |
| Processing speed | 0.69 |

**Table S2. Canonical correlation analysis between neighbourhood deprivation and modifiable lifestyle risk factors.**

|  | **Canonical loadings** |
| --- | --- |
| **Deprivation domains** |  |
| Income | 0.72 |
| Employment | 0.74 |
| Education Skills and Training | 0.71 |
| Health Deprivation and Disability | 0.59 |
| Barriers to Housing and Services | 0.40 |
| Crime | 0.46 |
| Living Environment | -0.19 |
| **Lifestyle risk factors** |  |
| Smoking | 0.02 |
| Alcohol | -0.47 |
| Unhealthy diet | 0.29 |
| Physical inactivity | 0.42 |
| Obesity | 0.40 |
| High blood pressure | 0.38 |
| High LDL cholesterol | -0.03 |
| Diabetes | -0.11 |
| Traumatic brain injury | 0.25 |
| Poor sleep | 0.48 |
| Depression | 0.20 |
| Social isolation | -0.01 |
| Hearing impairment | -0.19 |

**Table S3. Structural equation model parameter estimates**

|  | **Est** | **Std.Err** | **z-value** | **P(>\|z\|)** | **Std.lv** | **Std.all** |
| --- | --- | --- | --- | --- | --- | --- |
| **Latent variables** |  |  |  |  |  |  |
| Neighborhood Deprivation =~ |  |  |  |  |  |  |
| Income | 1 |  |  |  | 0.87 | 0.89 |
| Employment | 1.03 | 0.03 | 36.9 | <.001 | 0.9 | 0.91 |
| Education | 0.81 | 0.05 | 14.99 | <.001 | 0.7 | 0.71 |
| Health | 0.99 | 0.05 | 19.68 | <.001 | 0.86 | 0.87 |
| Crime | 0.55 | 0.06 | 9.43 | <.001 | 0.48 | 0.48 |
| Barriers: Housing & Services | 0.31 | 0.06 | 4.9 | <.001 | 0.27 | 0.27 |
| Living Environment | -0.12 | 0.09 | -1.41 | 0.16 | -0.11 | -0.11 |
| SVD =~ |  |  |  |  |  |  |
| White matter hyperintensities | 1 |  |  |  | 0.56 | 0.62 |
| Perivascular spaces | 0.85 | 0.17 | 4.95 | <.001 | 0.47 | 0.45 |
| Cerebral microbleeds | 0.09 | 0.05 | 1.86 | 0.06 | 0.05 | 0.13 |
| Lacunes | 0.13 | 0.04 | 3.1 | 0.002 | 0.07 | 0.24 |
| **Regressions** |  |  |  |  |  |  |
| SVD ~ |  |  |  |  |  |  |
| Neighborhood Deprivation | 0.11 | 0.05 | 2.54 | 0.011 | 0.18 | 0.18 |
| Age | 0.27 | 0.04 | 6.18 | <.001 | 0.49 | 0.48 |
| Sex | 0.35 | 0.09 | 3.94 | <.001 | 0.63 | 0.29 |
| Neighborhood Deprivation ~ |  |  |  |  |  |  |
| Education | -0.16 | 0.05 | -3.43 | 0.001 | -0.18 | -0.18 |

*Abbreviations: SVD = cerebral small vessel disease*

**p<.05, **p<.01, ***p<.001*

**Table S4. Serial mediation analysis with hypertensive arteriopathy subtype of SVD**

| **Direct Effects** | **Model 1** | **Model 2** | **Model 3** |
| --- | --- | --- | --- |
| Deprivation 🡪 Lifestyle | 0.11 [0.02, 0.19] | 0.11 [0.02, 0.19] | 0.11 [0.02, 0.18] |
| Deprivation 🡪 Lifestyle (Quadratic) | NA | 0.03 [-0.05, 0.11] | 0.03 [-0.05, 0.11] |
| Deprivation 🡪 HA-SVD | 0.23 [0.17, 0.28] | 0.22 [0.16, 0.28] | 0.22 [0.16, 0.28] |
| Deprivation 🡪 HA-SVD (Quadratic) | NA | 0.01 [-0.05, 0.08] | 0.01 [-0.06, 0.07] |
| Lifestyle 🡪 HA-SVD | 0.80 [0.73, 0.87] | 0.78 [0.71, 0.86] | 0.78 [0.70, 0.86] |
| HA-SVD 🡪 Cognition | 0.48 [0.35, 0.61] | 0.52 [0.36, 0.69] | 0.52 [0.36, 0.70] |
| Deprivation 🡪 Cognition | -0.05 [-0.14, 0.04] | -0.05 [-0.14, 0.04] | -0.05 [-0.14, 0.05] |
| Deprivation 🡪 Cognition (Quadratic) | NA | 0.09 [0.00, 0.17] | 0.09 [0.00, 0.18] |
| Deprivation × Lifestyle 🡪 Cognition | NA | NA | -0.11 [-0.30, 0.06] |
| Deprivation × HA-SVD 🡪 Cognition | NA | NA | 0.05 [-0.14, 0.22] |
| **Indirect Effects** |  |  |  |
| Deprivation 🡪 Lifestyle 🡪 HA-SVD 🡪 Cognition | 0.04 [0.01, 0.08] | 0.04 [0.01, 0.08] | 0.12 [0.07, 0.17] |
| Deprivation 🡪 HA-SVD 🡪 Cognition | 0.11 [0.07, 0.15] | 0.11 [0.07, 0.16] | 0.04 [0.01, 0.08] |
| **Total Effect** | 0.10 [0.01, 0.19] | 0.19 [0.06, 0.31] | 0.14 [-0.03, 0.29] |
| **Model Fit** |  |  |  |
| CFI | 1.000 | 1.000 | 0.969 |
| RMSEA | 0.000 | 0.000 | 0.08 |
| SRMR | 0.018 | 0.016 | 0.031 |

*Model 1: Linear structural equation model.*

*Model 2: Non-linear structural equation model.*

*Model 3: Non-linear structural equation model accounting for mediator-exposure interaction.*

*Note: Cognition was reverse coded for consistency across outcome measures, i.e., higher scores indicate poorer outcome. Parameter estimates are presented with 95% CI.*

*Abbreviations: HA-SVD = hypertensive arteriopathy subtype of cerebral small vessel disease, CI = confidence interval, CFI = Comparative Fit Index, RMSEA = Root Mean Square Error of Approximation, and SRMR = Standardized Root Mean Square Residual.*

**Table S5. Serial mediation analysis of CAA subtype of SVD**

| **Direct Effects** | **Model 1** | **Model 2** | **Model 3** |
| --- | --- | --- | --- |
| Deprivation 🡪 Lifestyle | 0.11 [0.03, 0.19] | 0.11 [0.03, 0.19] | 0.11 [0.03, 0.19] |
| Deprivation 🡪 Lifestyle (Quadratic) | NA | 0.05 [-0.03, 0.13] | 0.05 [-0.03, 0.13] |
| Deprivation 🡪 CAA-SVD | 0.05 [-0.02, 0.12] | 0.05 [-0.02, 0.12] | 0.05 [-0.02, 0.12] |
| Deprivation 🡪 CAA-SVD (Quadratic) | NA | -0.01 [-0.09, 0.08] | -0.01 [-0.09, 0.08] |
| Lifestyle 🡪 CAA-SVD | 0.66 [0.59, 0.74] | 0.66 [0.59, 0.74] | 0.66 [0.59, 0.74] |
| CAA-SVD 🡪 Cognition | 0.37 [0.23, 0.50] | 0.22 [0.09, 0.37] | 0.23 [0.09, 0.38] |
| Deprivation 🡪 Cognition | 0.05 [-0.04, 0.15] | 0.05 [-0.05, 0.14] | 0.05 [-0.04, 0.15] |
| Deprivation 🡪 Cognition (Quadratic) | NA | 0.09 [-0.01, 0.18] | 0.10 [0.00, 0.21] |
| Deprivation × Lifestyle 🡪 Cognition | NA | NA | -0.09 [-0.30, 0.08] |
| Deprivation × CAA-SVD 🡪 Cognition | NA | NA | 0.05 [-0.11, 0.22] |
| **Indirect Effects** |  |  |  |
| Deprivation 🡪 Lifestyle 🡪 CAA-SVD 🡪 Cognition | 0.03 [0.01, 0.05] | 0.01 [-0.01, 0.03] | 0.01 [-0.01, 0.03] |
| Deprivation 🡪 CAA-SVD 🡪 Cognition | 0.02 [-0.01, 0.05] | 0.02 [0, 0.04] | 0.02 [0, 0.04] |
| **Total Effect** | 0.10 [0.00, 0.19] | 0.18 [0.05, 0.32] | 0.16 [-0.02, 0.34] |
| **Model Fit** |  |  |  |
| CFI | 0.987 | 1.000 | 0.995 |
| RMSEA | 0.067 | 0.008 | 0.029 |
| SRMR | 0.028 | 0.020 | 0.025 |

*Model 1: Linear structural equation model.*

*Model 2: Non-linear structural equation model.*

*Model 3: Non-linear structural equation model accounting for mediator-exposure interaction.*

*Note: Cognition was reverse coded for consistency across outcome measures, i.e., higher scores indicate poorer outcome. Parameter estimates are presented with 95% CI.*

*Abbreviations: CAA-SVD = cerebral amyloid angiopathy subtype of cerebral small vessel disease, CI = confidence interval, CFI = Comparative Fit Index, RMSEA = Root Mean Square Error of Approximation, and SRMR = Standardized Root Mean Square Residual.*

**Table S6. Counterfactual-based decomposition of the effect of neighbourhood deprivation on cognition**

|  | **Mediation by Lifestyle** | **Mediation by SVD** |
| --- | --- | --- |
| Controlled Direct Effect (CDE)* | 0.07 [-0.03, 0.17] | 0.05 [-0.05, 0.15] |
| Pure Natural Direct Effect (PNDE) | 0.07 [-0.03, 0.17] | 0.05 [-0.05, 0.15] |
| Total Natural Indirect Effect (TNIE) | 0.03 [0.00, 0.06] | 0.04 [0.01, 0.08] |
| Total Natural Direct Effect (TNDE) | 0.07 [-0.03, 0.17] | 0.05 [-0.05, 0.16] |
| Pure Natural Indirect Effect (PNIE)* | 0.03 [0.00, 0.06] | 0.04 [0.01, 0.08] |
| Reference Interaction (INT_ref_)* | -0.002 [-0.101, 0.097] | -0.00 [-0.103, 0.102] |
| Mediated Interaction (INT_med_)* | -0.002 [-0.031, 0.026] | -0.000 [-0.033, 0.032] |
| Total Effect (TE) | 0.10 [0.00, 0.20] | 0.10 [0.00, 0.20] |
| Proportion Mediated (PM) | 0.28 [-0.08, 0.65] | 0.45 [-0.06, 0.97] |

*Note: Parameter estimates are presented with 95% CI.*

** The four basic components of VanderWeele’s four-way decomposition.^16^*

**Table S7. Parallel mediation analysis.**

| **Direct Effects** |  |
| --- | --- |
| Deprivation 🡪 Lifestyle | 0.11 [0.02, 0.19] |
| Lifestyle 🡪 Cognition | 0.21 [0.05, 0.37] |
| SVD 🡪 Cognition | 0.15 [0.02, 0.28] |
| Deprivation 🡪 Cognition | 0.05 [-0.04, 0.15] |
| Deprivation 🡪 SVD | 0.19 [0.09, 0.29] |
| **Indirect Effects** |  |
| Deprivation 🡪 Lifestyle 🡪 Cognition | 0.02 [0.00, 0.05] |
| Deprivation 🡪 SVD 🡪 Cognition | 0.03 [0.00, 0.06] |
| **Total Effect** | 0.10 [0.04, 0.20] |
| **Model Fit** |  |
| CFI | 0.622 |
| RMSEA | 0.346 |
| SRMR | 0.097 |

*Note: Cognition was reverse coded for consistency across outcome measures, i.e., higher scores indicate poorer outcome. Parameter estimates are presented with 95% CI.*

*Abbreviations: SVD = cerebral small vessel disease, CI = confidence interval, CFI = Comparative Fit Index, RMSEA = Root Mean Square Error of Approximation, and SRMR = Standardized Root Mean Square Residual.*

**Table S8. Sensitivity analysis of serial mediation analysis with data imputed under a missing not at random mechanism.**

| **Direct Effects** | **Model 1** | **Model 2** | **Model 3** |
| --- | --- | --- | --- |
| Deprivation 🡪 Lifestyle | 0.10 [0.02, 0.18] | 0.10 [0.02, 0.18] | 0.10 [0.02, 0.18] |
| Deprivation 🡪 Lifestyle (Quadratic) | NA | 0.03 [-0.05, 0.11] | 0.03 [-0.05, 0.11] |
| Deprivation 🡪 SVD | 0.09 [0.02, 0.17] | 0.09 [0.02, 0.17] | 0.09 [0.02, 0.17] |
| Deprivation 🡪 SVD (Quadratic) | NA | 0.01 [-0.07, 0.09] | 0.01 [-0.07, 0.09] |
| Lifestyle 🡪 SVD | 0.69 [0.59, 0.79] | 0.69 [0.59, 0.79] | 0.69 [0.59, 0.79] |
| SVD 🡪 Cognition | 0.30 [0.19, 0.41] | 0.16 [0.02, 0.29] | 0.17 [0.03, 0.29] |
| Deprivation 🡪 Cognition | 0.05 [-0.05, 0.14] | 0.05 [-0.05, 0.15] | 0.05 [-0.05, 0.15] |
| Deprivation 🡪 Cognition (Quadratic) | NA | 0.09 [0.00, 0.18] | 0.10 [0.01, 0.20] |
| Deprivation × Lifestyle 🡪 Cognition | NA | NA | -0.07 [-0.23, 0.08] |
| Deprivation × SVD 🡪 Cognition | NA | NA | 0.02 [-0.12, 0.15] |
| **Indirect Effects** |  |  |  |
| Deprivation 🡪 Lifestyle 🡪 SVD 🡪 Cognition | 0.02 [0.00, 0.04] | 0.01 [0.00, 0.03] | 0.02 [0.00, 0.04] |
| Deprivation 🡪 SVD 🡪 Cognition | 0.03 [0.00, 0.05] | 0.01 [0.00, 0.03] | 0.01 [0.00, 0.03] |
| **Total Effect** | 0.10 [0.00, 0.20] | 0.19 [0.05, 0.32] | 0.15 [-0.05, 0.32] |
| **Model Fit** |  |  |  |
| CFI | 0.986 | 0.999 | 0.948 |
| RMSEA | 0.067 | 0.018 | 0.089 |
| SRMR | 0.026 | 0.019 | 0.043 |

*Model 1: Linear structural equation model.*

*Model 2: Non-linear structural equation model.*

*Model 3: Non-linear structural equation model accounting for mediator-exposure interaction.*

*Note: Cognition was reverse coded for consistency across outcome measures, i.e., higher scores indicate poorer outcome. Parameter estimates are presented with 95% CI.*

*Abbreviations: SVD = cerebral small vessel disease, CI = confidence interval, CFI = Comparative Fit Index, RMSEA = Root Mean Square Error of Approximation, and SRMR = Standardized Root Mean Square Residual.*
